# TRIS–Egg Yolk Extender Attenuates Seminolipid and Sphingomyelin Loss in Chilled Canine Semen: An LC–HRMS Lipidomic and CASA Study

**DOI:** 10.3390/ani16152311

**Published:** 2026-07-27

**Authors:** Letizia Temerario, Enrico Liberato Cataldi, Giovanni Ventura, Nicola Antonio Martino, Maria Elena Dell’Aquila, Fabiola Tursi, Giovanni Michele Lacalandra, Giulio Guido Aiudi, Tommaso R. I. Cataldi

**Affiliations:** 1 Dipartimento di Bioscienze, Biotecnologie e Ambiente, University of Bari Aldo Moro, 70125 Bari, Italynicola.martino@uniba.it (N.A.M.);; 2 Dipartimento di Medicina Veterinaria, University of Bari Aldo Moro, 70125 Bari, Italygiovannimichele.lacalandra@uniba.it (G.M.L.);; 3 Dipartimento di Chimica and Centro di Ricerca Interdipartimentale SMART, University of Bari Aldo Moro, 70125 Bari, Italy; tommaso.cataldi@uniba.it; 4 Dipartimento di Farmacia-Scienze del Farmaco, University of Bari Aldo Moro, 70125 Bari, Italy; fabiola.tursi@gmail.com

**Keywords:** canine semen preservation, TRIS–egg yolk extender, chilled storage, seminolipids, sperm lipidomics, computer-assisted sperm analysis

## Abstract

This study combined lipidomic profiling and computer-assisted sperm analysis (CASA) to evaluate whether a TRIS–egg yolk extender preserves canine spermatozoa at both molecular and functional levels during 72 h of refrigerated storage. HILIC–ESI–HRMS was first used to distinguish sperm-derived lipids from extender-associated species. Lipid-focused feature filtering and multivariate analysis highlighted the seminolipid or sulfogalactosylglycerolipid SGG 32:0 as a major contributor to storage-related lipidomic variation. Together with three long-chain sphingomyelins, SM 46:5;O2, SM 48:5;O2, and SM 50:6;O2, SGG 32:0 was selected as a sperm-specific membrane marker. Targeted C30 reversed-phase LC–ESI–HRMS showed that these lipids progressively decreased during chilled storage, whereas their decline was significantly attenuated in extender-treated samples, with significant treatment × time interactions for all monitored species (*p* < 0.05). CASA confirmed the functional relevance of these molecular changes: in extender-treated semen, total motility was preserved during the first 24 h, whereas more evident impairment occurred at 48–72 h. Overall, these findings indicate that TRIS–egg yolk extender helps preserve canine semen by slowing the loss of sperm-specific membrane lipids and maintaining motility during chilled storage. SGG 32:0 may represent a candidate lipid marker for monitoring sperm membrane preservation.

## Simple Summary

The preservation of canine semen during chilled storage is essential for artificial insemination, but spermatozoa progressively undergo membrane destabilisation, oxidative damage, and loss of motility. Semen extenders containing TRIS and egg yolk are commonly used to reduce this deterioration, although their protective effects at the molecular level are not fully understood. In this study, we combined advanced lipid analysis with computer-assisted evaluation of sperm movement to investigate how chilled storage affects sperm membranes and whether the extender can limit this damage. We identified specific membrane lipids that are naturally present in sperm cells and monitored their changes during 72 h of storage. These lipids gradually decreased over time, but their loss was significantly reduced when the extender was used. This molecular preservation was accompanied by better maintenance of sperm motility during the early stages of refrigeration. Among the analysed compounds, one sperm-specific lipid emerged as a promising indicator of membrane preservation. These findings improve our understanding of how semen extenders protect canine sperm and may help develop better strategies for semen preservation and assisted reproduction in dogs.

## 1. Introduction

Assisted reproductive technologies are now widely used in canine breeding, both to optimise genetic selection in commercial breeds and to support the conservation of rare or endangered genotypes. Among the available approaches, artificial insemination with chilled semen represents a practical and widely adopted strategy, as it allows semen to be transported across long distances without the infrastructure required for cryogenic storage. When appropriately extended, canine semen can retain fertilising capacity for several days at 4 °C, although sperm quality progressively declines during storage. Compared with cryopreservation, chilled storage represents a milder procedure, but it still exposes spermatozoa to cold-induced membrane stress, reactive oxygen species formation, oxidative damage and gradual loss of motility and fertilising capacity [1,2,3,4,5,6,7].

Semen extenders are therefore essential to preserve sperm function during refrigerated storage. Formulations based on tris(hydroxymethyl)aminomethane (TRIS), citric acid and fructose, supplemented with egg yolk, are among the most used extenders for canine semen. Their protective effect has been mainly attributed to the low-density lipoprotein fraction of egg yolk, which can stabilise the sperm plasma membrane and limit the loss of cholesterol and phospholipids during storage. In addition, egg-yolk lipids may contribute to the maintenance of membrane integrity, thereby supporting sperm viability and motility under low-temperature conditions [8,9,10]. The sperm plasma membrane has a highly specialised lipid composition that is closely linked to sperm function, in which choline-containing lipids, including phosphatidylcholines (PCs) and sphingomyelins (SMs), are enriched in the outer leaflet. The high content of long-chain polyunsaturated fatty acids provides the membrane flexibility required for capacitation, motility and sperm–oocyte interaction, but also makes spermatozoa particularly susceptible to lipid peroxidation [11,12]. Among sperm lipids, seminolipids, also known as sulfogalactosylglycerolipids (SGGs), are of particular interest. Seminolipid is a sulphated glyceroglycolipid highly enriched in testicular germ cells and mature spermatozoa, where it contributes to membrane organisation, capacitation-related remodelling and sperm–egg interaction. Disruption of seminolipid biosynthesis causes male infertility in knockout models, and altered seminolipid levels have been associated with impaired sperm parameters in humans [13,14,15,16,17,18,19]. SMs are another important class of sperm membrane lipids that contribute to membrane order and stability. In several mammalian species, sperm sphingomyelins include very-long-chain molecular species that are uncommon in somatic cells and may be relevant to the structural properties of the sperm membrane [11,20,21]. In canine spermatozoa, previous MALDI-MS studies have shown a phospholipid profile dominated by PCs, with a detectable SM contribution, and have suggested associations between membrane lipid composition and sperm motility [22,23]. However, to the best of our knowledge, LC-MS-based lipidomic changes occurring in chilled canine semen during a biologically relevant storage period remain largely unexplored.

The present study was designed to address this gap by combining liquid chromatography coupled with electrospray ionisation and high-resolution mass spectrometry (LC–ESI–HRMS), lipidomic profiling and computer-assisted sperm analysis. The aim was to identify semen-specific lipid markers, monitor their temporal evolution in canine semen stored at 4 °C in the presence and absence of a TRIS–egg yolk extender, and evaluate whether molecular preservation of sperm membrane lipids is associated with the maintenance of sperm motility during refrigerated storage.

## 2. Materials and Methods

### 2.1. Chemicals

LC–MS grade water, acetonitrile, methanol and isopropanol, HPLC-grade chloroform, formic acid and ammonium acetate were obtained from Merck (Milan, Italy). The deuterated lipid internal standard mixture EquiSPLASH^®^ LIPIDOMIX^®^ was purchased from Avanti Polar Lipids (Alabaster, AL, USA). Tris(hydroxymethyl)aminomethane, citric acid monohydrate, D-fructose, and penicillin–streptomycin were obtained from Merck (Milan, Italy).

### 2.2. Ethics Committee

The study was approved by the Ethics Committee of the Department of Veterinary Medicine, University of Bari Aldo Moro, Italy (Approval no. 09/23, Prot. no. 1226-III/13). All procedures were performed according to good veterinary practice and applicable national regulations on animal welfare. Written informed owner consent was obtained before enrolment.

### 2.3. Animals

The study was conducted at the Obstetrics, Gynaecology and Andrology Clinic of the Veterinary Hospital, Department of Veterinary Medicine, University of Bari Aldo Moro, Italy. Candidate dogs underwent clinical and ultrasonographic examination to assess general and reproductive health and to exclude reproductive tract disorders. Serum canine prostatic specific esterase (CPSE), a marker commonly used for the evaluation of prostatic status in dogs, was measured from venous blood collected from the cephalic vein [24]. Dogs were included only if they showed normal CPSE values, no clinical or ultrasonographic evidence of reproductive disease, physiological basal semen parameters and a positive response to semen collection by digital manipulation. Six healthy dogs met these criteria and were enrolled in the study. Their signalment and basal semen-quality parameters are reported in Supplementary Table S1.

### 2.4. Semen Collection and Refrigeration

Semen was collected by manual stimulation with the aid of an artificial vagina, after olfactory stimulation with oestrous pheromones from bitches in oestrous. The ejaculate was separated into urethral, sperm-rich, and prostatic fractions according to their sequential emission during collection. Only the sperm-rich fraction was used for the study. For each dog, the sperm-rich fraction was divided into aliquots assigned to untreated semen (S) or semen diluted with extender (S+E). Extended samples were prepared by mixing one volume of semen with three volumes of extender, corresponding to a semen ratio of 1:3 (*v*/*v*) and to a four-fold final dilution. Given the relatively homogeneous initial sperm concentrations among donors, ranging from 364 to 401 × 10^6^ spermatozoa/mL (Table S1), the final concentrations after dilution were comparable and approximately ranged from 91 to 100 × 10^6^ spermatozoa/mL. The extender consisted of tris(hydroxymethyl)aminomethane (TRIS; 3.028 g), citric acid monohydrate (1.78 g) and D-fructose (1.25 g) dissolved in 100 mL of ultrapure water, supplemented with 20% egg yolk and penicillin–streptomycin (1 mg/mL), according to Teixeira et al. [25]. Samples were stored at 4 °C and analysed after 3, 24, 48 and 72 h of refrigeration.

### 2.5. Sample Preparation and Extraction of Lipids

Untreated semen samples, extender-only controls and semen–extender mixtures were processed for lipidomic analysis. For each donor and storage time, aliquots were maintained at 4 °C until extraction. For semen-derived samples, 200 µL were used for lipid extraction. Extender-only controls were processed using 800 µL, corresponding to the semen volume present in the 1:3 semen/extender dilution scheme, to account for the lipid contribution of the extender matrix. Each sample was supplemented with 200 µL of methanol containing the EquiSPLASH^®^ internal standard mixture at a concentration of 1.0 ppm. Lipids were extracted according to the Bligh and Dyer [26] procedure. After phase separation, the lower organic phase was carefully collected and evaporated to dryness under a gentle nitrogen stream. The lipid residue was reconstituted in 1.0 mL of the appropriate injection solvent before LC–MS analysis.

Quality control (QC) samples were prepared by pooling equal volumes of all reconstituted extracts, according to established lipidomics quality-assurance protocols [27]. QC samples were used to monitor analytical stability and to support quality-control-based robust LOESS signal correction (QC-RLSC). Three solvent blanks were injected at the beginning of each batch, followed by five consecutive QC injections for column conditioning. QC samples were then injected every five study samples throughout the analytical sequence and twice at the end of each batch. The complete injection order is reported in Table S2.

### 2.6. Lipid Annotation, Data Preprocessing and Feature Filtering

Lipid annotations were assigned in accordance with the LIPID MAPS classification framework [28,29]. For ether lipids, the nomenclature proposed by Koelmel et al. [30] was adopted. For the HILIC dataset used for exploratory lipidomic profiling, raw files were converted to mzXML format using MSConvert from ProteoWizard 3.0.26202. Data preprocessing was performed in XCMS Online 3.7.1, based on the XCMS platform for LC–MS data processing and nonlinear peak alignment [31]. Feature detection was carried out in negative ion mode using the matchedFilter algorithm with the following settings: full width at half maximum, 30 s; signal-to-noise threshold, 10; step size, 0.1 *m*/*z*; number of steps, 2; maximum number of peaks, 10; and mzdiff, 0.01. Retention-time correction was performed using the obiwarp algorithm. Features were grouped across samples using the density method with bandwidth 5, mzwid 0.013, minfrac 0.15 and minsamp 1, followed by peak filling to recover missing signals.

Signal drift correction was performed using quality-control-based robust LOESS signal correction (QC-RLSC), based on pooled QC injections distributed throughout the analytical batch. Only features detected in at least 70% of QC injections and exhibiting a relative standard deviation below 20% in QC samples were retained for downstream analysis [32].

The dataset was further curated using a custom MATLAB R2025b (MathWorks, Natick, MA, USA) algorithm aimed at retaining lipid-related features only. Detected signals were matched against a predefined list of exact masses of target lipid species using a mass tolerance of ±5 ppm. In addition, retention times were constrained within predefined chromatographic windows derived from previous lipidomic studies acquired under identical chromatographic conditions, thereby improving selectivity for biologically plausible lipid species and reducing false non-lipid background signals [33].

### 2.7. Instrumentation and Operating Conditions

High-resolution mass spectrometric analyses were carried out using an Ultimate 3000 UHPLC system coupled to a Q-Exactive hybrid quadrupole–Orbitrap mass spectrometer (Thermo Scientific, Waltham, MA, USA) equipped with a heated electrospray ionisation source and an HCD collision cell for MS/MS experiments. The main source and ion-transmission parameters were set as follows: sheath gas, 35 a.u.; auxiliary gas, 15 a.u.; spray voltage, −3.5 kV in negative ion mode; capillary temperature, 320 °C; and S-Lens RF level, 60 a.u. Tandem MS experiments were performed using a normalised collision energy of 35%. Precursor ions were isolated within a 1 *m*/*z* window, selecting the monoisotopic peak before fragmentation. Hydrophilic interaction liquid chromatography (HILIC) was performed at 25 ± 1 °C using an Ascentis Express HILIC column (150 × 2.1 mm, 2.7 µm particle size, Merck, Milan, Italy) equipped with a matching guard cartridge (5 × 2.1 mm). The flow rate was 300 µL/min, and 5 µL of lipid extract were injected. The binary mobile-phase system consisted of solvent A, water containing 2.5 mmol/L ammonium acetate and 0.1% formic acid, and solvent B, acetonitrile with 0.1% formic acid. The gradient started at 97% B and was linearly decreased to 88% B within 5 min, followed by an isocratic step at 88% B from 5 to 10 min. The composition was further reduced to 81% B between 10 and 11 min and then gradually decreased to 70% B by 20 min. A final linear decrease to 50% B was applied from 20 to 22 min and maintained isocratic until 28 min. The system was returned to the initial conditions over 2 min and allowed to equilibrate for 5 min before the next injection.

Reversed-phase separations were performed on an Accucore™ C30 RP-MS column (150 × 2.1 mm, 2.6 µm core–shell particles) equipped with a compatible guard cartridge (5 × 2.1 mm, Thermo Scientific, Waltham, MA, USA). The column temperature was maintained at 30 °C, and the flow rate was 300 µL/min. The mobile phases consisted of solvent A, acetonitrile/water 60:40, *v*/*v*, and solvent B, isopropanol/acetonitrile/water 90:10:1, *v*/*v*/*v*, both supplemented with 10 mM ammonium formate and 0.1% formic acid. The gradient program began at 35% B and was held for 3 min, followed by a linear increase to 50% B at 7 min and to 65% B at 15 min. The composition was maintained at 65% B until 20 min and then increased to 90% B at 30 min and to 99% B at 32 min. The high-organic condition was sustained for 40 min to ensure elution of strongly retained lipid species. The system was then returned to initial conditions and equilibrated for 5 min before the subsequent run.

### 2.8. Selection of Lipid Species for Time-Course and Statistical Analysis

Lipid species were initially annotated based on accurate *m*/*z* and retention time in canine semen samples. To select lipids for longitudinal comparison between untreated and extender-treated conditions, candidate species were prioritised according to: (i) high signal intensity in semen QC samples, ensuring robust quantification; (ii) consistent detection across the experimental dataset; and (iii) minimal contribution from the extender matrix, assessed by analysing extender-only samples. Based on these criteria, one seminolipid, SGG 32:0, and three sphingomyelins, SM 46:5;O2, SM 48:5;O2 and SM 50:6;O2, were selected for downstream time-course modelling. Treatment was coded as untreated semen (S) or extender-treated semen (S+E). Because repeated measurements were obtained from the same donors across multiple storage times, lipid data were analysed using linear mixed-effects models to account for within-donor correlation. For each lipid species, normalised intensity was modelled as a function of treatment, storage time and their interaction, using dog identity as a random intercept. The model was fitted using the MATLAB fitlme function [34] according to the following specification: *Value ~ Treatment × Time + (1|Dog).* Treatment and storage time were included as fixed effects, whereas donor identity was modelled as a random intercept. Storage time was treated as a categorical factor to avoid assumptions about linear or monotonic temporal trends. The treatment × time interaction was considered the main effect of interest, as it tests whether extender exposure modifies the time-dependent pattern of lipid abundance rather than introducing a constant offset across all time points. Fixed effects were evaluated by analysis of variance on the fitted mixed models. To account for multiple testing across lipid species, *p*-values for the treatment × time interaction were adjusted using the Benjamini–Hochberg false-discovery-rate procedure [35]. When a significant interaction was observed, post hoc contrasts comparing extender-treated and untreated samples were performed separately at each storage time and adjusted for multiple comparisons using the Holm–Bonferroni method [36].

### 2.9. Computer-Assisted Sperm Analysis (CASA)

Semen kinetic parameters were assessed using the IVOS-Sperm CASA system, version 12.3, Hamilton Thorne, MA, USA, as previously described for canine semen [37,38] at 3, 24, 48 and 72 h of refrigerated storage at 4 °C, corresponding to the same time points used for lipidomic analysis. CASA analysis was performed on semen samples extended with TRIS–egg yolk extender (S+E), while untreated semen (S) was assessed at the initial time point as a reference condition. The CASA software (IVOS 12.3) was set up using parameters specific to canine semen. According to the manufacturer’s instructions, a 3 µL drop of each sample was diluted twofold in the corresponding prostatic fraction and loaded onto a 20 µm depth, four-chamber Leja slide. The slide was placed in the dedicated microscope chamber and allowed to settle for a few seconds before analysis. Five randomly selected non-consecutive microscopic fields were scanned for each sample. The following parameters were evaluated: sperm concentration, total motility, progressive motility, average path velocity (VAP), straight-line velocity (VSL), curvilinear velocity (VCL), amplitude of lateral head displacement (ALH), beat-cross frequency (BCF), straightness (STR) and linearity (LIN). In addition, according to the low VAP cut-off (LVV) and medium VAP cut-off (MVV), spermatozoa were classified into four speed classes: fast spermatozoa, medium-speed spermatozoa, slow spermatozoa and static spermatozoa, representing the fraction of immotile cells during analysis.

### 2.10. Statistical Analysis

CASA-derived sperm kinetic parameters were analysed using repeated-measures one-way ANOVA followed by Tukey’s multiple-comparison test, because the same dogs were evaluated across multiple storage times. Data were analysed using GraphPad Prism, version 5.03, San Diego, CA, USA. Differences were considered statistically significant at *p* < 0.05.

## 3. Results and Discussion

### 3.1. HILIC-ESI-HRMS Lipidomic Profiling of Canine Semen and Egg-Yolk Extender

Hydrophilic interaction liquid chromatography (HILIC) coupled to high-resolution mass spectrometry (HRMS) with electrospray ionisation (ESI) was first used to profile the polar lipid composition of canine semen and of the egg-yolk extender. The approach served to identify semen-specific lipids suitable as molecular markers of sperm membrane preservation during chilled storage exploiting the head-group-governed retention of HILIC separations [39,40,41,42].

**Figure 1:**
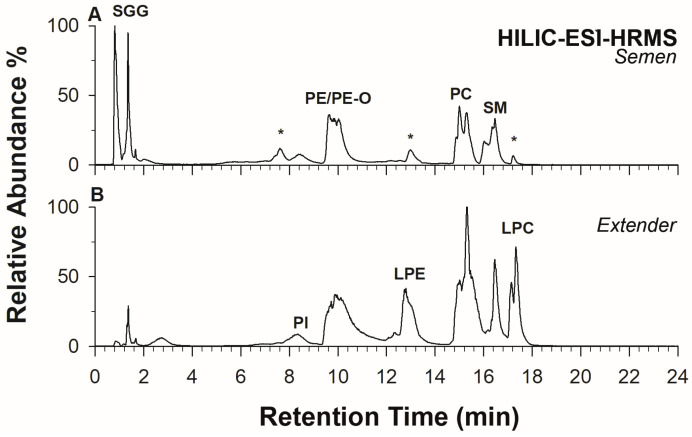
Representative total ion current chromatograms by HILIC-ESI-HRMS acquired in negative ion mode from canine semen (**A**) and extender-only extract (**B**). Major lipid-class regions are indicated, including phosphatidylethanolamines (PEs), phosphatidylcholines (PCs), sphingomyelins (SMs) and phosphatidylinositols (PIs). Several lipid classes were detected in both matrices, whereas a distinctive early-eluting feature close to the void volume was observed only in semen and was absent from the extender, suggesting the presence of highly polar sperm-specific lipid species. Asterisked peaks mainly correspond to internal standard signals.

HILIC–ESI–HRMS total ion current (TIC) chromatograms acquired in negative ion mode showed clear qualitative differences between semen and extender-only extracts (Figure 1A,B). Several lipid-related chromatographic regions were common to both matrices, including phosphatidylethanolamines (PEs), phosphatidylcholines (PCs) and sphingomyelins (SMs), consistent with the broad occurrence of these lipid classes in biological membranes and in egg-yolk-derived formulations. Conversely, phosphatidylinositols and several phosphatidylcholine species were more prominent in the extender chromatogram, confirming the strong contribution of egg-yolk lipids to the overall lipid background of extended semen samples. Notably, a distinctive chromatographic band was observed exclusively in semen samples and was absent from the extender-only control.

**Figure 2:**
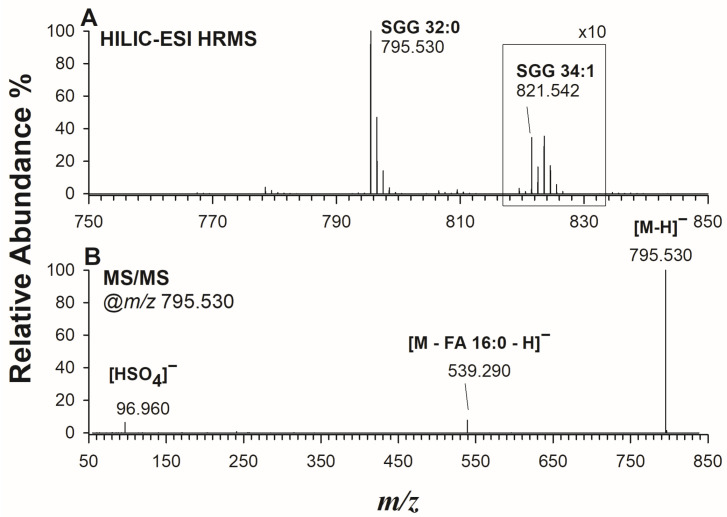
HRMS and MS/MS identification of SGG 32:0 in canine semen. (**A**) HRMS spectrum acquired in negative ion mode from the early-eluting HILIC region. The dominant ion at *m*/*z* 795.530 was assigned to the deprotonated molecule [M–H]^−^ of SGG 32:0, a seminolipid highly enriched in spermatozoa and testicular tissue. (**B**) Tandem mass spectrum of the precursor ion at *m*/*z* 795.530. The diagnostic fragment at *m*/*z* 96.960, corresponding to HSO_4_^−^, confirms the presence of a sulphated head group, while the fragment at *m*/*z* 539.290 is consistent with the neutral loss of a 16:0 fatty acyl chain, supporting the assignment as SGG 16:0/16:0.

This band eluted close to the void volume of the HILIC separation, indicating poor retention under these conditions and suggesting the presence of highly polar sperm-associated lipid species. High-resolution mass spectra acquired in this region revealed ions consistent with seminolipids, also known as sulfogalactosylglycerolipids (SGGs), a lipid class highly enriched in spermatozoa and testicular tissue and closely associated with male reproductive function [14]. The dominant ion at *m*/*z* 795.530 was assigned to the deprotonated molecule [M–H]^−^ of SGG 32:0, while minor ions at *m*/*z* 823.561 and 821.542 were tentatively attributed to SGG 34:0 and SGG 34:1 (see Figure 2A), respectively.

**Figure 3:**
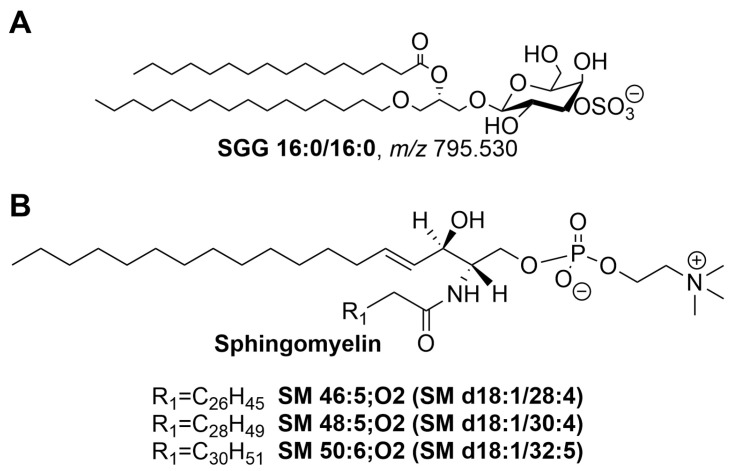
Proposed structures of sperm-derived lipid markers selected for targeted analysis. (**A**) Proposed structure of SGG 32:0, assigned as 1-O-hexadecyl-2-O-palmitoyl-3-O-(3-O-sulfo-β-D-galactopyranosyl)-sn-glycerol (SGG 16:0/16:0), detected in negative ion mode as [M–H]^−^ at *m*/*z* 795.530. SGG is a sperm-enriched sulfoglycolipid involved in membrane organisation and sperm–egg interaction. (**B**) Proposed long-chain sphingomyelin species (28, 30, and 32 carbon atoms) were detected selectively in semen and were absent from the extender-only matrix. In negative ion mode, these zwitterionic lipids were mainly observed as formate (HCOO^−^) adducts, including SM 46:5;O2 at *m*/*z* 907.690, SM 48:5;O2 at *m*/*z* 935.722 and SM 50:6;O2 at *m*/*z* 961.738. Double-bond positions and geometries were not assigned.

The identity of SGG 32:0 was further supported by tandem mass spectrometry (see Figure 2B). The MS/MS spectrum of the precursor ion at *m*/*z* 795.530 showed the diagnostic hydrogen sulphate fragment at *m*/*z* 96.960, confirming the presence of a sulphated head group, together with a fragment at *m*/*z* 539.290 consistent with the neutral loss of a 16:0 fatty acyl chain. Overall, the accurate mass and fragmentation behaviour supported the assignment of this species as SGG 16:0/16:0, and the proposed structure is shown in Figure 3A. The selective occurrence of this lipid in semen, together with its established association with sperm membrane organisation and sperm–egg interaction, indicated that SGG 32:0 could serve as a robust endogenous marker of sperm membrane integrity [14,43].

### 3.2. Selection of Sperm-Derived Lipid Markers for Monitoring Membrane Preservation

To investigate the evolution of the semen lipidome during chilled storage, the HILIC–ESI–HRMS data were processed using a lipid-focused metabolomic workflow. XCMS was first used to extract all detectable LC–MS signals, generating a feature table in which each feature was defined by an accurate *m*/*z* value, a retention time and an intensity across samples. This table was then filtered to retain only features plausibly attributable to lipid species, based on accurate mass matching within 5 ppm, class-specific HILIC retention windows and the most common ion form observed in negative ion mode (Supplementary Table S3). This strategy was adopted to reduce the risk of overinterpreting non-lipid features and to improve the biological reliability of the dataset, in line with current recommendations for lipidomics annotation and reporting [28,29,30]. This filtering restricted the dataset to biologically meaningful lipid classes, including ceramides (CERs), phosphatidylcholines (PCs), phosphatidylinositols (PIs), phosphatidylethanolamines (PEs), sphingomyelins (SMs), lysophospholipids (LPLs) and seminolipids (SLs). Before multivariate analysis, intensities were normalised to the total ion signal of each extract to reduce variability associated with sample loading and extraction efficiency. Only features showing a relative standard deviation below 20% in pooled QC samples were retained, ensuring that subsequent interpretation was based on analytically reproducible signals. The use of pooled QC samples and RSD-based filtering is consistent with established recommendations for quality assurance in LC–MS metabolomics and lipidomics workflows [27]. After filtering, 307 lipid-related features were included in the final dataset.

**Figure 4:**
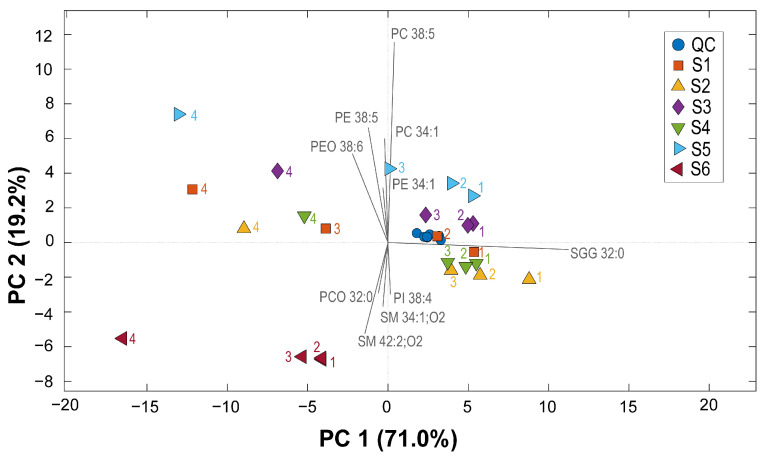
PCA biplot obtained from lipid-related features retained after QC-based filtering. Tight clustering of pooled QC samples indicates analytical stability and confirms the robustness of the lipidomic workflow, in agreement with recommended quality-control practices for LC–MS metabolomics. Biological samples show a storage-time-associated displacement along PC1, indicating progressive remodelling of the semen lipidome during chilled storage. The loading contribution of SGG 32:0 highlights the relevance of seminolipids as major drivers of lipidomic variation.

Principal component analysis (PCA) of semen samples analysed in the absence of extender showed tight clustering of QC injections, confirming the analytical stability of the HILIC–MS workflow (Figure 4).

Biological samples were distributed according to both donor-related variability and storage time. Notably, a progressive shift along PC1 was observed with increasing storage duration, indicating systematic remodelling of the semen lipidome during chilled storage. Inspection of the loading plot showed that this temporal displacement was largely associated with seminolipid species, particularly SGG 32:0, which contributed strongly to the variance along PC1. Because the study aimed to monitor sperm-derived membrane components rather than lipid species introduced by the extender, the extender-only matrix was processed using the same workflow. This comparison showed that many phospholipids detected in semen were also present in the extender, as expected from the lipid-rich composition of egg yolk. These shared species were not suitable for evaluating sperm-specific lipid preservation in S+E samples, because their signal could not be unambiguously assigned to the sperm membrane.

A limited number of PCs, PEs and related lyso- or ether-linked species were found to be exclusive to semen, but their low signal intensity reduced their suitability as robust quantitative markers. Several long-chain ceramides were also detected only in semen; however, their low abundance and the distribution of their signal across multiple ion forms, including [M–H]^−^, [M+Cl]^−^ and [M+HCOO]^−^, limited their quantitative reliability. This behaviour is consistent with previous observations on the complex ionisation patterns of acidic and neutral glycosphingolipids in negative ion mode [44]. In contrast, a group of long-chain SMs was selectively detected in semen and showed adequate signal intensity. These species were chromatographically associated with an early-eluting shoulder in the sphingomyelin region, consistent with the behaviour of long-chain sphingomyelins under HILIC conditions. Although HILIC retention is primarily controlled by polar head-group interactions, hydrophobicity, chain length and unsaturation can modulate elution within a lipid class [45].

Based on these observations, SGG 32:0 and three long-chain sphingomyelins, namely SM 46:5;O2, SM 48:5;O2 and SM 50:6;O2, were selected as sperm-derived lipid markers for targeted monitoring. The proposed structures of the selected long-chain sphingomyelins are shown in Figure 3B. Their absence from the extender-only matrix made them particularly suitable for evaluating whether the TRIS–egg yolk extender could attenuate the loss of endogenous sperm membrane lipids during chilled storage. This selection is also biologically meaningful, since sperm membranes are highly specialised lipid environments and sphingolipids are known to contribute to membrane organisation, stability and susceptibility to storage-induced damage [14,46]. However, the poor HILIC retention of SGG and the limited species-level resolution of HILIC required a complementary reversed-phase strategy for reliable quantitative assessment [40,41,42].

### 3.3. Targeted C30 RPLC–ESI–HRMS Analysis Reveals Extender-Mediated Preservation of Sperm-Specific Lipids

**Figure 5:**
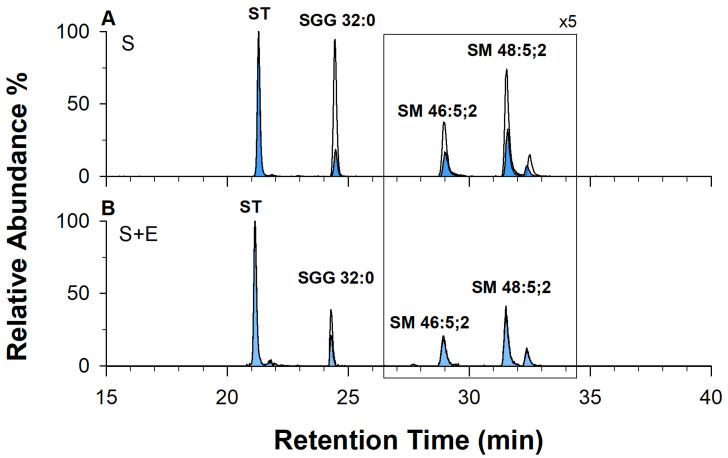
RPLC–ESI–HRMS extracted ion chromatograms of sperm-specific lipid markers during chilled storage. Representative overlaid extracted ion chromatograms of SGG 32:0, SM 46:5;O2, SM 48:5;O2 and SM 50:6;O2 obtained from a selected donor by targeted C30 RPLC–ESI–HRMS. (**A**) Untreated semen (S) and (**B**) extender-treated semen (S+E) are compared after 3 h and 72 h of chilled storage. Peak areas were normalised to the internal standard (I.S.). SGG 32:0 and the selected long-chain sphingomyelins showed a progressive signal decrease over time, with a more pronounced reduction in untreated semen. The sphingomyelin region is magnified to improve visualisation of lower-intensity species.

To quantify the selected sperm-derived lipid markers with improved chromatographic resolution, a targeted C30 reversed-phase LC–ESI–HRMS method was applied. Unlike HILIC, which primarily separates lipids according to polar head-group chemistry, C30 reversed-phase chromatography provides enhanced discrimination according to acyl chain length, unsaturation and hydrophobicity, making it particularly suitable for lipid species-level analysis [40,41,42]. This was essential for selectively monitoring SGG 32:0 and long-chain sphingomyelins in the complex lipid background generated by the egg-yolk extender. Under C30 reversed-phase conditions, SGG 32:0 eluted before the sphingomyelin species, consistent with its lower overall hydrophobicity. The three long-chain sphingomyelins eluted subsequently, according to their increasing chain length and unsaturation. Representative extracted ion chromatograms from a selected donor are shown in Figure 5, comparing untreated semen and extender-treated semen at 3 h and 72 h of chilled storage.

The target species were clearly detectable in both experimental groups, although a progressive signal decrease was observed over storage time, particularly in untreated samples. For quantitative comparison across donors, peak areas were normalised to the internal standard and then expressed relative to the first storage time point for each donor, thereby reducing the effect of inter-individual baseline variability. All four monitored lipid species showed a significant time-dependent decrease during chilled storage. However, this decline was consistently more pronounced in untreated semen than in extender-treated samples, suggesting that the TRIS–egg yolk extender attenuated the loss of endogenous sperm membrane lipids.

Linear mixed-effects modelling showed a significant effect of storage time for all monitored lipid species and, more importantly, a significant treatment × time interaction after Benjamini–Hochberg correction (Table 1).

The main effect of treatment was not significant for any lipid species and is therefore not reported in the table. This indicates that the extender did not produce a constant difference in lipid abundance across all storage times but rather modified the temporal trajectory of lipid loss. Therefore, the protective effect of the extender should be interpreted as a slowing of sperm-specific lipid depletion during storage.

**Figure 6:**
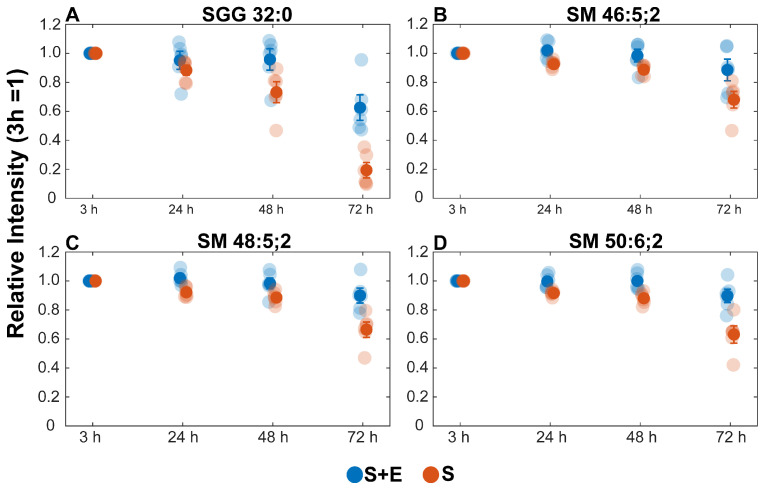
Relative abundance of sperm-specific lipids in untreated semen (S) and extender-treated semen (S+E) during chilled storage: (**A**) SGG 32:0; (**B**) SM 46:5;O2; (**C**) SM 48:5;O2; (**D**) SM 50:6;O2. Lipid abundances were calculated from internal-standard-normalised peak areas and expressed relative to the first storage time point (3 h) for each donor to minimise inter-individual baseline variability. Individual points represent single dogs (*n* = 6) and summary symbols indicate the mean ± SEM. All lipid species decreased over time, consistent with storage-induced membrane lipid deterioration and oxidative stress, although the decline was more pronounced in untreated semen. Linear mixed-effects modelling showed a significant treatment × time interaction for all monitored lipids after Benjamini–Hochberg correction (q < 0.05), indicating that the extender modulates lipid-loss kinetics during storage.

Applying this model to each lipid species, the protective effect was particularly evident for SGG 32:0, whose abundance decreased markedly in untreated semen but was better preserved in the presence of the extender (Figure 6). 

This finding is biologically relevant because seminolipids are sperm-enriched sulfoglycolipids involved in membrane organisation, lipid raft architecture and sperm–egg interaction [14]. Altered seminolipid metabolism has also been associated with impaired reproductive function, supporting the relevance of SGG as a molecular marker of sperm membrane status [43]. Therefore, the progressive loss of SGG during chilled storage may reflect deterioration of functionally important membrane domains, whereas its partial preservation in extender-treated samples suggests a molecular basis for the protective action of the TRIS–egg yolk formulation.

A similar behaviour was observed for the long-chain sphingomyelins SM 46:5;O2, SM 48:5;O2 and SM 50:6;O2. These species are expected to contribute to sperm membrane architecture and stability. Previous studies have shown that phospholipid and sphingolipid species enriched in polyunsaturated or very-long-chain residues can be selectively affected by semen storage and cryopreservation, supporting their use as informative markers of membrane deterioration [46]. Their partial preservation in extender-treated semen therefore strengthens the interpretation that TRIS–egg yolk extender helps maintain sperm membrane organisation during chilled storage.

Taken together, the targeted lipidomic data indicate that chilled storage induces a progressive depletion of sperm-specific membrane lipids, including SGG 32:0 and selected long-chain sphingomyelins, which is significantly slowed by the TRIS–egg yolk extender.

### 3.4. CASA Analysis Confirms Functional Preservation of Sperm Motility During Chilled Storage

**Figure 7:**
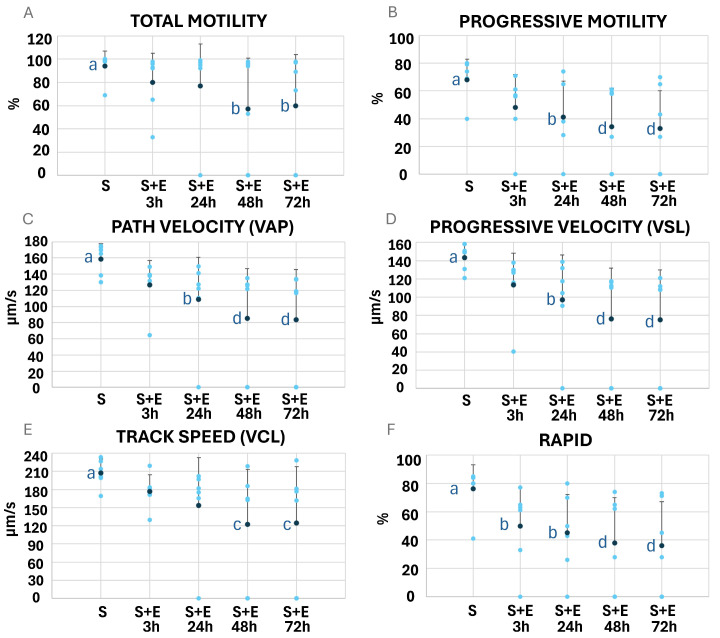
Computer-assisted sperm analysis was performed on fresh semen and on semen stored at 4 °C in TRIS–egg yolk extender for up to 72 h. The figure reports six representative functional parameters: total motility (**A**), progressive motility (**B**), average path velocity (VAP; (**C**)), straight-line velocity (VSL; (**D**)), curvilinear velocity (VCL; (**E**)), and percentage of fast spermatozoa (**F**). These parameters were selected to summarise the main functional effects of refrigeration on sperm movement. Data are shown as mean ± SEM; individual points represent single dogs (*n* = 6). Statistical differences among time points were assessed by repeated-measures one-way ANOVA followed by Tukey’s multiple-comparison test, considering the dog as the repeated subject. Significance levels are indicated as *p* < 0.05 (a,b), *p* < 0.01 (a,c) and *p* < 0.001 (a,d). Additional CASA parameters are reported in Supplementary Figure S1. S, untreated semen at the initial time point; S+E, semen diluted with TRIS–egg yolk extender and stored at 4 °C for the indicated time.

Because sperm motility depends critically on the integrity, fluidity and organisation of the plasma membrane, computer-assisted sperm analysis (CASA) was used to evaluate whether the lipid-protective effect observed by LC–MS was associated with preservation of sperm function. CASA provides an objective quantitative assessment of sperm movement by measuring both global motility parameters and more detailed descriptors of sperm trajectory. In the main figure, we focused on six representative parameters: total motility, progressive motility, average path velocity (VAP), straight-line velocity (VSL), curvilinear velocity (VCL), and the percentage of fast spermatozoa (Figure 7).

These parameters were selected because they provide a concise and biologically meaningful overview of sperm functional competence during refrigerated storage. Additional CASA descriptors, including amplitude of lateral head displacement (ALH), beat-cross frequency (BCF), straightness (STR), linearity (LIN), and the distribution of medium, slow and static spermatozoa, are reported in the Supplementary Materials (Figure S1).

In extender-treated samples, total motility was preserved during the early phase of refrigeration, remaining comparable to fresh semen at 3 h and 24 h, whereas a significant decline became evident after 48 h and 72 h of storage (*p* < 0.05). Progressive motility was more sensitive to storage, decreasing already after 24 h (*p* < 0.05) and showing a further reduction at 48 h and 72 h (*p* < 0.001) (Figure 7A,B). This behaviour is consistent with previous evidence that chilled storage progressively compromises sperm quality through cold shock, oxidative stress, reactive oxygen species formation and lipid peroxidation [6,7,9,12].

The three velocity-related parameters provided further insight into the quality of sperm movement during storage. Average path velocity, which describes the mean velocity along a smoothed sperm trajectory, and straight-line velocity, which measures the velocity along the direct path from the starting point to the endpoint, were both maintained at 3 h but decreased from 24 h onward. This reduction was moderate at 24 h (*p* < 0.05) and became more pronounced at 48 h and 72 h (*p* < 0.001). By contrast, curvilinear velocity, which represents the actual velocity along the real sperm trajectory and is therefore influenced by lateral head movement, remained comparable to fresh semen up to 24 h and declined only after 48 h and 72 h (*p* < 0.01) (Figure 7C–E). These findings indicate that refrigerated spermatozoa initially retain motile activity but progressively lose movement efficiency and forward-directed progression during prolonged storage.

The proportion of fast spermatozoa was included in the main figure as an intuitive descriptor of functional deterioration. Fast spermatozoa decreased rapidly during refrigeration, with a significant reduction already at 3 h and 24 h (*p* < 0.05) and a more pronounced decrease at 48 h and 72 h (*p* < 0.001) (Figure 7F). This decline indicates that refrigeration does not simply reduce the number of motile cells but progressively shifts the sperm population away from the high-performance motile fraction. The remaining speed-class parameters, reported in the Supplementary Materials, showed that medium- and slow-speed sperm populations were broadly comparable to fresh samples across the analysed time points, whereas static spermatozoa increased after refrigeration, reaching significance from 3 h onward (*p* < 0.05). Similarly, the supplementary trajectory descriptors confirmed that the amplitude of lateral head displacement was not significantly affected by refrigeration, whereas beat-cross frequency decreased at 48 h and 72 h (*p* < 0.05), and straightness and linearity were preserved up to 24 h before declining at later storage times. Overall, the CASA results indicate that extender-treated spermatozoa retained a relatively stable functional profile during the first day of refrigerated storage, followed by a progressive but not abrupt decline during prolonged refrigeration. This functional trend agrees with the general understanding that storage at low temperature progressively reduces sperm performance [1,2,4].

Importantly, these functional results are consistent with the lipidomic data. The decrease in motility and kinetic parameters over time parallels the progressive loss of sperm-specific membrane lipids, particularly SGG 32:0 and long-chain sphingomyelins. However, in the presence of the TRIS–egg yolk extender, both lipid depletion and functional deterioration were attenuated. The preservation of motility in extender-treated samples is consistent with the established protective role of egg-yolk-based extenders against low-temperature-induced membrane damage [25,47]. Thus, CASA does not merely confirm that motility declines during refrigeration, which is expected; rather, it provides functional evidence that the lipid-preserving effect observed in extender-treated samples has physiological relevance. The combined LC–MS and CASA results support a coherent model in which chilled storage progressively destabilises sperm membranes, an effect mitigated by the TRIS–egg yolk extender. This study has some limitations that define the scope of the present findings. The analysis was based on a limited number of donors (*n* = 6), and confirmation in larger cohorts would strengthen the conclusions. Lipid changes were monitored on a semi-quantitative basis, using internal-standard normalisation to track temporal trends; absolute quantification of the sperm-specific markers, and of SGG 32:0, was beyond the present scope, as isotopically labelled standards for this seminolipid are not yet available. Finally, the study assessed molecular and motility markers but did not include reproductive outcomes; the relationship between lipid preservation and actual fertilising capacity therefore remains to be established.

## 4. Conclusions

This study demonstrates that TRIS–egg yolk extender helps preserve canine semen during chilled storage by attenuating both molecular and functional markers of sperm deterioration. HILIC–ESI–HRMS enabled discrimination between endogenous sperm-derived lipids and lipid species introduced by the egg-yolk extender, allowing the identification of SGG 32:0 and selected long-chain sphingomyelins as semen-specific markers of sperm membrane composition. Targeted RPLC–ESI–HRMS showed that these lipids progressively decreased during chilled storage, consistent with cold-induced membrane remodelling and lipid deterioration. However, their decline was significantly attenuated in extender-treated samples, indicating that the extender actively modulates the kinetics of sperm membrane lipid loss rather than simply supplementing the semen matrix. CASA analysis provided functional support for the lipidomic findings. Although refrigeration progressively affected motility and kinetic parameters, extender-treated samples retained total motility and key kinetic descriptors during the early storage period, with more evident deterioration only after prolonged refrigeration. The agreement between lipidomic and CASA data is consistent with a model in which TRIS–egg yolk extender helps preserve sperm membrane organisation, which may in turn support the maintenance of motility during chilled storage. Overall, SGG 32:0 emerges as a promising sperm-specific lipid marker for monitoring membrane preservation in canine semen, although its validation as a reliable biomarker will require dedicated studies. Extending this approach to cryopreserved semen, together with absolute quantification of SGG 32:0, would be a natural next step in this direction.

## Figures and Tables

**Table 1:** Linear mixed-effects analysis of sperm-specific lipid temporal dynamics during chilled storage. Normalised peak areas of each lipid species were analysed using a linear mixed-effects model including treatment, storage time and their interaction as fixed effects, with dog identity included as a random effect. Time tests whether lipid abundance changes during chilled storage. Treatment × Time tests whether the temporal trajectory differs between untreated semen and semen diluted with TRIS–egg yolk extender. The *q*-values correspond to Benjamini–Hochberg false-discovery-rate correction applied to the treatment × time interaction. A significant treatment × time interaction indicates that extender treatment modified the kinetics of lipid loss during storage.

Lipid	*p* Time	*p* Treatment × Time	*q* Treatment × Time
SGG 32:0	6.18 × 10^−7^	6.37 × 10^−5^	1.27 × 10^−4^
SM 46:5;O2	1.23 × 10^−2^	1.38 × 10^−2^	1.38 × 10^−2^
SM 48:5;O2	5.40 × 10^−3^	2.57 × 10^−4^	3.43 × 10^−4^
SM 50:6;O2	5.80 × 10^−3^	1.44 × 10^−5^	5.75 × 10^−5^

Treatment refers to untreated semen (S) or semen diluted with TRIS–egg yolk extender (S+E). *p*-values indicate the statistical significance of each fixed effect in the linear mixed-effects model. Time tests whether lipid abundance changes during refrigerated storage, whereas Treatment × Time tests whether the temporal profile differs between untreated semen (S) and semen diluted with TRIS–egg yolk extender (S+E). *q*-values are Benjamini–Hochberg false-discovery-rate-adjusted *p*-values for the Treatment × Time interaction, used to account for multiple testing across lipid species.

## Data Availability

The data presented in this study are available on request.

## Supplementary Materials

The following supporting information can be downloaded at: https://www.mdpi.com/article/10.3390/ani16152311/s1, Table S1: Clinical information of enrolled dogs in the study; Table S2: Injection order for semen analysis; Table S3: Lipid species detected in canine semen (QC sample) and egg yolk-based extender by HILIC-ESI-HRMS untargeted lipidomic profiling; Figure S1: Computer-assisted sperm analysis was performed on fresh semen and on semen stored at 4 °C in TRIS–egg yolk extender for up to 72 h.

## Abbreviations

The following abbreviations are used in this manuscript:

CASA	Computer-assisted sperm analysis
ESI	Electrospray ionisation
HILIC	Hydrophilic interaction liquid chromatography
HRMS	High-resolution mass spectrometry
QC	Quality control
RPLC	Reversed-phase liquid chromatography
SGG	Sulfogalactosylglycerolipid (seminolipid)
SM	Sphingomyelin
TRIS	Tris(hydroxymethyl)aminomethane
