# Supplementary material for: TRIS–Egg Yolk Extender Attenuates Seminolipid and Sphingomyelin Loss in Chilled Canine Semen: An LC–HRMS Lipidomic and CASA Study
Source: Animals (Basel). 2026 Jul 27;16(15):2311. doi: 10.3390/ani16152311 (PMC13463695; doi:10.3390/ani16152311)
Supplement: Supplementary file 1 [file animals-16-02311-s001.zip › animals-4417669-supplementary.pdf]

Type of the Paper: Article

TRIS–Egg Yolk Extender Attenuates Seminolipid and Sphingomyelin Loss in Chilled Canine Semen: An LC–HRMS Lipidomic and CASA Study

Letizia Temerario [1], Enrico Liberato Cataldi [2], Giovanni Ventura [3,*], Nicola Antonio Martino [1], Maria Elena Dell'Aquila [1], Fabiola Tursi [4], Giovanni Michele Lacalandra [2], Giulio Guido Aiudi [2,†], and Tommaso R.I. Cataldi[3,†]

[1] Dipartimento di Bioscienze, Biotecnologie and Ambiente, University of Bari Aldo Moro, Italy
[2] Dipartimento di Medicina Veterinaria, University of Bari Aldo Moro, Italy
[3] Dipartimento di Chimica and Centro di Ricerca Interdipartimentale SMART, University of Bari Aldo Moro, Italy
[4] Dipartimento di Farmacia - Scienze del Farmaco, University of Bari Aldo Moro, Italy
* Correspondence: giovanni.ventura@uniba.it
† These authors contributed equally to this work.

Keywords: Canine semen preservation, TRIS–egg yolk extender, chilled storage, seminolipids, sperm lipidomics, computer-assisted sperm analysis

Number of Figures: 1 (S1)

Number of Tables: 3 (S1, S2, S3)

Table S1. Clinical information of enrolled dogs in the study.

Dog names	Dog breeds	Age (years)	Body weight (kg)	Semen concentration (*10^6 cell/mL*)
1 Darko	Border Collie	4	23.4	395
2 Luke	English Bulldog	3.5	24	377
3 Don Paolo	German Shepherd	4.2	32.3	386
4 Bisonte	German Shepherd	3	35	401
5 Sasa	German Shepherd	4	36.2	364
6 Byron	Belgian Shepherd	4	34	398

Overall, the animals represented a relatively homogeneous group in terms of reproductive age and initial semen quality. Semen concentration values showed limited inter-individual variability, confirming the suitability of the ejaculates for comparative analyses. This relative homogeneity is relevant for the interpretation of the experimental results, as it minimises potential confounding effects related to major differences in age, body weight, or baseline seminal concentration.

Table S2. Injection order for semen analysis. Each sample is labelled as #G, where # denotes the individual donor and G the storage day. Samples from the same donor share the same colour code. QC: quality control sample.

Inj Order	Sample	Inj Order	Sample
1	Blank	20	#1_G4
2	QC_1	21	QC_8
3	QC_2	22	#2_G1
4	QC_3	23	#2_G4
5	QC_4	24	#5_G2
6	QC_5	25	#6_G1
7	#5_G3	26	QC_9
8	#4_G1	27	#6_G2
9	#2_G3	28	#1_G3
10	#4_G4	29	#4_G3
11	QC_6	30	#3_G1
12	#2_G2	31	QC_10
13	#3_G4	32	#1_G1
14	#1_G2	33	#5_G4
15	#6_G3	34	#4_G2
16	QC_7	35	#6_G4
17	#5_G1	36	QC_11
18	#3_G3	37	QC_12
19	#3_G2	38	Blank

The injection sequence was designed to monitor analytical stability throughout the LC-MS analysis. Blank injections were placed at the beginning and end of the batch to check for background signals and potential carryover, while QC samples were repeatedly injected across the sequence to assess instrumental reproducibility and signal stability over time. Study samples were distributed across the batch according to donor and storage day, reducing the risk that analytical drift could systematically affect a specific biological group or time point.

Table S3. Lipid species detected in canine semen (QC sample) and egg yolk-based extender by HILIC-ESI-HRMS untargeted lipidomic profiling. For each species, the following information is reported: experimental retention time (RT, min), lipid class, sum composition, ion attribution, and theoretical m/z. Semen and Extender columns report the peak area of the corresponding species in each matrix; CN (%) denotes the class-normalised relative intensity, calculated as the peak area of each species divided by the sum of peak areas of all species belonging to the same lipid class within the same matrix, expressed as a percentage. For species detected exclusively in one matrix, the corresponding values in the other matrix are reported as 0. Where multiple adducts of the same sum composition were detected in the extender, the highest peak area was retained.

mz	RT	Class	SumComp	Attribution	Semen	Semen CN(%)	Extender	Extender CN (%)
767.4985	0.8	SGG	SGG 30:0	[SGG 30:0 -H]-	3.87E+06	0.51	0	0
819.5298	0.8	SGG	SGG 34:2	[SGG 34:2 -H]-	2.09E+06	0.28	0	0
793.5141	0.8	SGG	SGG 32:1	[SGG 32:1 -H]-	3.35E+06	0.44	0	0
821.5454	0.8	SGG	SGG 34:1	[SGG 34:1 -H]-	2.36E+07	3.14	0	0
795.5298	0.9	SGG	SGG 32:0	[SGG 32:0 -H]-	6.96E+08	92.33	0	0
823.5611	0.9	SGG	SGG 34:0	[SGG 34:0 -H]-	2.49E+07	3.3	0	0
794.6668	1.3	Cer	Cer 50:7;2	[Cer 50:7;2 +HCO_2]-	1.79E+05	0.23	0	0
812.6774	1.3	Cer	Cer 50:6;3	[Cer 50:6;3 +HCO_2]-	3.86E+05	0.5	0	0
796.6824	1.3	Cer	Cer 50:6;2	[Cer 50:6;2 +HCO_2]-	1.05E+06	1.35	0	0
784.6461	1.3	Cer	Cer 48:6;3	[Cer 48:6;3 +HCO_2]-	6.34E+05	0.82	0	0
768.6511	1.3	Cer	Cer 48:6;2	[Cer 48:6;2 +HCO_2]-	5.62E+05	0.72	0	0
798.6981	1.3	Cer	Cer 50:5;2	[Cer 50:5;2 +HCO_2]-	4.34E+05	0.56	0	0
744.6511	1.3	Cer	Cer 46:4;2	[Cer 46:4;2 +HCO_2]-	2.21E+05	0.28	0	0
772.6613	1.3	Cer	Cer 52:9;2	[Cer 52:9;2 -H]-	6.93E+05	0.89	0	0
824.7137	1.3	Cer	Cer 52:6;2	[Cer 52:6;2 +HCO_2]-	7.25E+05	0.93	0	0
770.6668	1.3	Cer	Cer 48:5;2	[Cer 48:5;2 +HCO_2]-	3.70E+06	4.76	0	0
732.6067	1.3	Cer	Cer 46:5;2	[Cer 46:5;2 +Cl]-	8.91E+06	11.47	0	0
734.5729	1.3	Cer	Cer 46:9;2	[Cer 46:9;2 +HCO_2]-	3.79E+06	4.88	0	0
692.6198	1.3	Cer	Cer 42:2;2	[Cer 42:2;2 +HCO_2]-	3.64E+06	4.68	17411666	27.61
706.591	1.3	Cer	Cer 44:4;2	[Cer 44:4;2 +Cl]-	5.04E+05	0.65	0	0
668.5987	1.3	Cer	Cer 44:5;2	[Cer 44:5;2 -H]-	7.39E+05	0.95	0	0
666.6042	1.4	Cer	Cer 40:1;2	[Cer 40:1;2 +HCO_2]-	4.18E+06	5.38	13678165	21.69
694.6355	1.4	Cer	Cer 42:1;2	[Cer 42:1;2 +HCO_2]-	6.80E+06	8.75	13124520	20.81
638.5729	1.4	Cer	Cer 38:1;2	[Cer 38:1;2 +HCO_2]-	4.86E+06	6.26	1493768	2.37
654.5597	1.4	Cer	Cer 40:2;2	[Cer 40:2;2 +Cl]-	1.29E+06	1.66	0	0
538.5205	1.4	Cer	Cer 34:0;2	[Cer 34:0;2 -H]-	3.93E+05	0.51	2386148	3.78
608.5048	1.4	Cer	Cer 40:7;2	[Cer 40:7;2 -H]-	2.00E+05	0.26	0	0
610.5416	1.4	Cer	Cer 36:1;2	[Cer 36:1;2 +HCO_2]-	4.93E+06	6.34	1898577	3.01
546.4658	1.4	Cer	Cer 32:0;2	[Cer 32:0;2 +Cl]-	1.73E+05	0.22	0	0
718.6144	1.4	Cer	Cer 48:8;2	[Cer 48:8;2 -H]-	5.74E+05	0.74	0	0
848.6621	1.4	HexCer	HexCer 44:4;3	[HexCer 44:4;3 -H]-	1.44E+06	4.99	0	0
700.6016	1.4	Cer	Cer 42:1;3	[Cer 42:1;3 +Cl]-	6.93E+06	8.92	5034772	7.98
836.6388	1.4	HexCer	HexCer 40:0;3	[HexCer 40:0;3 +Cl]-	3.31E+06	11.5	0	0

508.4735	1.4	Cer	Cer 32:1;2	[Cer 32:1;2 -H]-	1.54E+05	0.2	0	0
820.6308	1.4	HexCer	HexCer 42:4;3	[HexCer 42:4;3 -H]-	6.52E+05	2.26	0	0
890.6857	1.4	HexCer	HexCer 44:1;3	[HexCer 44:1;3 +Cl]-	4.49E+05	1.56	0	0
582.5103	1.4	Cer	Cer 34:1;2	[Cer 34:1;2 +HCO_2]-	2.50E+06	3.22	7248675	11.49
674.586	1.4	Cer	Cer 40:0;3	[Cer 40:0;3 +Cl]-	2.27E+06	2.92	0	0
800.6329	1.4	Cer	Cer 50:7;3	[Cer 50:7;3 +Cl]-	3.55E+05	0.46	0	0
722.6668	1.4	Cer	Cer 44:1;2	[Cer 44:1;2 +HCO_2]-	2.82E+05	0.36	236904.6	0.38
702.6173	1.4	Cer	Cer 42:0;3	[Cer 42:0;3 +Cl]-	6.67E+06	8.58	0	0
626.5365	1.4	Cer	Cer 36:1;3	[Cer 36:1;3 +HCO_2]-	8.34E+05	1.07	211548.9	0.34
774.677	1.4	Cer	Cer 52:8;2	[Cer 52:8;2 -H]-	2.94E+05	0.38	0	0
552.4997	1.4	Cer	Cer 34:1;3	[Cer 34:1;3 -H]-	1.29E+06	1.65	211866.2	0.34
740.6693	1.4	Cer	Cer 46:1;2	[Cer 46:1;2 +Cl]-	3.20E+05	0.41	0	0
692.6562	1.4	Cer	Cer 44:1;3	[Cer 44:1;3 -H]-	7.32E+04	0.09	0	0
554.5154	1.5	Cer	Cer 34:0;3	[Cer 34:0;3 -H]-	4.91E+05	0.63	133248.5	0.21
766.693	1.5	Cer	Cer 46:1;3	[Cer 46:1;3 +HCO_2]-	8.99E+04	0.12	0	0
806.7607	1.5	Cer	Cer 50:1;2	[Cer 50:1;2 +HCO_2]-	3.87E+04	0.05	0	0
588.4189	1.6	Cer	Cer 36:7;2	[Cer 36:7;2 +Cl]-	5.56E+06	7.16	0	0
790.5969	2.1	HexCer	HexCer 38:1;2	[HexCer 38:1;2 +Cl]-	1.72E+06	5.96	0	0
818.6282	2.1	HexCer	HexCer 40:1;2	[HexCer 40:1;2 +Cl]-	1.96E+06	6.8	1957910	7.27
864.6701	2.1	HexCer	HexCer 42:0;3	[HexCer 42:0;3 +Cl]-	6.71E+06	23.31	0	0
844.6439	2.1	HexCer	HexCer 42:2;2	[HexCer 42:2;2 +Cl]-	1.46E+06	5.07	268907.5	1
808.6075	2.1	HexCer	HexCer 38:0;3	[HexCer 38:0;3 +Cl]-	9.32E+05	3.24	0	0
762.5656	2.1	HexCer	HexCer 36:1;2	[HexCer 36:1;2 +Cl]-	1.52E+06	5.29	1407918	5.22
846.6465	2.2	HexCer	HexCer 44:5;3	[HexCer 44:5;3 -H]-	1.05E+06	3.65	1002984	3.72
738.5526	2.2	HexCer	HexCer 36:3;3	[HexCer 36:3;3 -H]-	3.26E+05	1.13	381540.4	1.42
736.55	2.2	HexCer	HexCer 34:0;2	[HexCer 34:0;2 +Cl]-	8.74E+05	3.04	637919.6	2.37
734.5343	2.2	HexCer	HexCer 34:1;2	[HexCer 34:1;2 +Cl]-	1.87E+06	6.51	8644497	32.08
888.6701	2.3	HexCer	HexCer 44:2;3	[HexCer 44:2;3 +Cl]-	2.57E+05	0.89	0	0
860.6388	2.3	HexCer	HexCer 42:2;3	[HexCer 42:2;3 +Cl]-	1.22E+06	4.25	12103096	44.91
878.6515	2.3	HexCer	HexCer 48:9;2	[HexCer 48:9;2 -H]-	1.52E+06	5.28	0	0
880.6672	2.3	HexCer	HexCer 48:8;2	[HexCer 48:8;2 -H]-	1.52E+06	5.29	543833.6	2.02
896.5871	5.8	Hex2Cer	Hex2Cer 34:1;2	[Hex2Cer 34:1;2 +Cl]-	7.64E+05	4.64	0	0
1006.697	6.2	Hex2Cer	Hex2Cer 42:2;2	[Hex2Cer 42:2;2 +Cl]-	2.73E+06	16.58	0	0
1010.715	6.2	Hex2Cer	Hex2Cer 44:4;3	[Hex2Cer 44:4;3 -H]-	2.72E+06	16.55	0	0
1008.712	6.2	Hex2Cer	Hex2Cer 42:1;2	[Hex2Cer 42:1;2 +Cl]-	5.94E+06	36.1	134843.6	100
982.6836	6.2	Hex2Cer	Hex2Cer 42:4;3	[Hex2Cer 42:4;3 -H]-	8.16E+05	4.96	0	0
980.681	6.2	Hex2Cer	Hex2Cer 40:1;2	[Hex2Cer 40:1;2 +Cl]-	1.78E+06	10.83	0	0
952.6497	6.3	Hex2Cer	Hex2Cer 38:1;2	[Hex2Cer 38:1;2 +Cl]-	5.13E+05	3.11	0	0
996.676	6.6	Hex2Cer	Hex2Cer 40:1;3	[Hex2Cer 40:1;3 +Cl]-	9.09E+05	5.52	0	0
968.6447	6.7	Hex2Cer	Hex2Cer 38:1;3	[Hex2Cer 38:1;3 +Cl]-	2.82E+05	1.71	0	0
771.5182	6.7	PG	PG 36:3	[PG 36:3 -H]-	2.77E+05	1.14	982949.5	3.38
771.5182	7.6	PG	PG 36:3	[PG 36:3 -H]-	4.17E+05	1.72	982949.5	3.38

773.5338	7.6	PG	PG 36:2	[PG 36:2 -H]-	2.07E+06	8.53	16677076	57.31
775.5495	7.6	PG	PG 36:1	[PG 36:1 -H]-	2.51E+06	10.36	6004986	20.63
747.5182	7.6	PG	PG 34:1	[PG 34:1 -H]-	1.76E+07	72.78	4453549	15.3
721.5025	7.7	PG	PG 32:0	[PG 32:0 -H]-	1.33E+06	5.47	0	0
909.5499	8.2	PI	PI 40:6	[PI 40:6 -H]-	1.19E+06	1	2624208	0.97
911.5655	8.2	PI	PI 40:5	[PI 40:5 -H]-	7.32E+06	6.14	5342668	1.97
883.5342	8.2	PI	PI 38:5	[PI 38:5 -H]-	2.47E+06	2.07	2677511	0.99
913.5812	8.3	PI	PI 40:4	[PI 40:4 -H]-	4.32E+06	3.63	6678840	2.46
885.5499	8.3	PI	PI 38:4	[PI 38:4 -H]-	1.82E+07	15.28	1.3E+08	47.87
887.5655	8.3	PI	PI 38:3	[PI 38:3 -H]-	6.71E+06	5.63	59735287	22.03
888.5549	8.3	PE	PE 48:13	[PE 48:13 -H]-	3.04E+06	0.55	0	0
857.5186	8.3	PI	PI 36:4	[PI 36:4 -H]-	1.05E+06	0.88	5243462	1.93
889.5812	8.3	PI	PI 38:2	[PI 38:2 -H]-	5.03E+06	4.22	0	0
859.5342	8.4	PI	PI 36:3	[PI 36:3 -H]-	4.90E+05	0.41	6791510	2.5
861.5499	8.4	PI	PI 36:2	[PI 36:2 -H]-	3.45E+06	2.89	36239323	13.36
833.5186	8.4	PI	PI 34:2	[PI 34:2 -H]-	1.13E+06	0.95	9941119	3.67
863.5655	8.4	PI	PI 36:1	[PI 36:1 -H]-	7.53E+06	6.32	0	0
835.5342	8.4	PI	PI 34:1	[PI 34:1 -H]-	5.31E+06	4.45	4626447	1.71
837.5499	8.5	PI	PI 34:0	[PI 34:0 -H]-	1.91E+07	16.01	557566.9	0.21
809.5186	8.5	PI	PI 32:0	[PI 32:0 -H]-	8.85E+06	7.42	0	0
865.5812	8.5	PI	PI 36:0	[PI 36:0 -H]-	2.71E+07	22.71	931330.3	0.34
976.6801	9.5	PE	PE 54:11	[PE 54:11 -H]-	2.89E+05	0.05	0	0
978.6957	9.5	PE	PE 54:10	[PE 54:10 -H]-	5.87E+05	0.11	0	0
838.5392	9.5	PE	PE 44:10	[PE 44:10 -H]-	1.50E+06	0.27	983883.8	0.06
950.6644	9.5	PE	PE 52:10	[PE 52:10 -H]-	9.33E+05	0.17	0	0
952.6801	9.5	PE	PE 52:9	[PE 52:9 -H]-	1.56E+06	0.28	0	0
926.6644	9.5	PE	PE 50:8	[PE 50:8 -H]-	6.60E+05	0.12	0	0
836.5236	9.5	PE	PE 44:11	[PE 44:11 -H]-	8.20E+05	0.15	0	0
924.6488	9.5	PE	PE 50:9	[PE 50:9 -H]-	5.78E+05	0.1	0	0
812.5236	9.5	PE	PE 42:9	[PE 42:9 -H]-	2.33E+06	0.42	456572.4	0.03
770.513	9.6	PEO	PEO 40:9	[PEO 40:9 -H]-	2.33E+05	0.05	0	0
842.5705	9.6	PE	PE 44:8	[PE 44:8 -H]-	8.15E+05	0.15	1993950	0.13
772.5287	9.6	PEO	PEO 40:8	[PEO 40:8 -H]-	6.18E+06	1.32	13553415	4.57
774.5443	9.6	PEO	PEO 40:7	[PEO 40:7 -H]-	1.93E+07	4.13	87334702	29.42
776.56	9.6	PEO	PEO 40:6	[PEO 40:6 -H]-	1.57E+07	3.37	36181893	12.19
816.5549	9.6	PE	PE 42:7	[PE 42:7 -H]-	6.40E+05	0.12	1426557	0.09
748.5287	9.6	PEO	PEO 38:6	[PEO 38:6 -H]-	2.07E+08	44.16	18519139	6.24
746.513	9.6	PEO	PEO 38:7	[PEO 38:7 -H]-	5.11E+07	10.93	30925807	10.42
818.5705	9.6	PE	PE 42:6	[PE 42:6 -H]-	1.26E+06	0.23	884338.8	0.06
820.5862	9.7	PE	PE 42:5	[PE 42:5 -H]-	1.29E+06	0.23	523254.9	0.03
788.5236	9.7	PE	PE 40:7	[PE 40:7 -H]-	4.21E+06	0.76	8087710	0.52
790.5392	9.7	PE	PE 40:6	[PE 40:6 -H]-	2.78E+07	5.03	63936078	4.15

722.513	9.7	PEO	PEO 36:5	[PEO 36:5 -H]-	1.38E+07	2.94	1622716	0.55
720.4974	9.7	PEO	PEO 36:6	[PEO 36:6 -H]-	1.42E+06	0.3	0	0
792.5549	9.7	PE	PE 40:5	[PE 40:5 -H]-	5.59E+07	10.12	27988102	1.82
764.5236	9.8	PE	PE 38:5	[PE 38:5 -H]-	1.39E+08	25.1	41824930	2.71
762.5079	9.8	PE	PE 38:6	[PE 38:6 -H]-	5.33E+07	9.64	51367556	3.33
694.4817	9.8	PEO	PEO 34:5	[PEO 34:5 -H]-	5.39E+05	0.12	0	0
766.5392	9.8	PE	PE 38:4	[PE 38:4 -H]-	8.65E+07	15.65	2.75E+08	17.81
724.5287	9.8	PEO	PEO 36:4	[PEO 36:4 -H]-	4.22E+06	0.9	1751273	0.59
738.5079	9.9	PE	PE 36:4	[PE 36:4 -H]-	2.10E+07	3.8	57728426	3.74
750.5443	9.9	PEO	PEO 38:5	[PEO 38:5 -H]-	1.18E+08	25.26	7991303	2.69
726.5443	9.9	PEO	PEO 36:3	[PEO 36:3 -H]-	8.39E+06	1.79	19467864	6.56
696.4974	9.9	PEO	PEO 34:4	[PEO 34:4 -H]-	1.21E+05	0.03	0	0
698.513	9.9	PEO	PEO 34:3	[PEO 34:3 -H]-	2.21E+06	0.47	2529085	0.85
700.5287	10.0	PEO	PEO 34:2	[PEO 34:2 -H]-	6.57E+06	1.41	18189988	6.13
756.5913	10.0	PEO	PEO 38:2	[PEO 38:2 -H]-	1.36E+06	0.29	760640.3	0.26
770.5705	10.0	PE	PE 38:2	[PE 38:2 -H]-	3.13E+06	0.57	2974213	0.19
672.4974	10.0	PEO	PEO 32:2	[PEO 32:2 -H]-	1.33E+06	0.28	0	0
728.56	10.0	PEO	PEO 36:2	[PEO 36:2 -H]-	6.75E+06	1.44	46506976	15.67
740.5236	10.0	PE	PE 36:3	[PE 36:3 -H]-	8.00E+06	1.45	40850932	2.65
772.5862	10.0	PE	PE 38:1	[PE 38:1 -H]-	1.68E+06	0.31	2401224	0.16
742.5392	10.1	PE	PE 36:2	[PE 36:2 -H]-	2.27E+07	4.11	2.66E+08	17.23
744.5549	10.1	PE	PE 36:1	[PE 36:1 -H]-	2.06E+07	3.73	2.54E+08	16.46
714.5079	10.1	PE	PE 34:2	[PE 34:2 -H]-	2.51E+07	4.54	1.59E+08	10.29
716.5236	10.1	PE	PE 34:1	[PE 34:1 -H]-	5.85E+07	10.59	2.78E+08	18.03
686.4766	10.1	PE	PE 32:2	[PE 32:2 -H]-	3.67E+05	0.07	392340.4	0.03
646.4817	10.1	PEO	PEO 30:1	[PEO 30:1 -H]-	1.95E+05	0.04	0	0
688.4923	10.1	PE	PE 32:1	[PE 32:1 -H]-	3.52E+06	0.64	5622557	0.36
674.513	10.2	PEO	PEO 32:1	[PEO 32:1 -H]-	9.97E+05	0.21	753436.3	0.25
770.513	10.2	PEO	PEO 40:9	[PEO 40:9 -H]-	6.95E+05	0.15	0	0
702.5443	10.2	PEO	PEO 34:1	[PEO 34:1 -H]-	1.85E+06	0.4	10768936	3.63
690.5079	10.2	PE	PE 32:0	[PE 32:0 -H]-	4.41E+06	0.8	1675213	0.11
662.4766	10.3	PE	PE 30:0	[PE 30:0 -H]-	7.67E+05	0.14	0	0
526.2939	12.2	LPE	LPE 22:5	[LPE 22:5 -H]-	8.70E+06	43.02	7269009	0.61
528.3096	12.2	LPE	LPE 22:4	[LPE 22:4 -H]-	7.71E+05	3.81	986769.1	0.08
504.3096	12.2	LPE	LPE 20:2	[LPE 20:2 -H]-	1.53E+05	0.76	0	0
524.2783	12.2	LPE	LPE 22:6	[LPE 22:6 -H]-	2.74E+06	13.56	19287819	1.61
506.3252	12.3	LPE	LPE 20:1	[LPE 20:1 -H]-	2.38E+05	1.18	0	0
464.3146	12.3	LPEO	LPEO 18:1	[LPEO 18:1 -H]-	1.50E+06	35.84	40880961	54.87
500.2783	12.3	LPE	LPE 20:4	[LPE 20:4 -H]-	1.85E+06	9.14	33156401	2.76
502.2939	12.4	LPE	LPE 20:3	[LPE 20:3 -H]-	1.69E+05	0.84	2354891	0.2
462.299	12.4	LPEO	LPEO 18:2	[LPEO 18:2 -H]-	1.17E+06	27.93	7305226	9.8
478.2939	12.5	LPE	LPE 18:1	[LPE 18:1 -H]-	7.97E+05	3.94	1.3E+08	10.87

436.2833	12.6	LPEO	LPEO 16:1	[LPEO 16:1 -H]-	1.52E+06	36.23	26324764	35.33
476.2783	12.7	LPE	LPE 18:2	[LPE 18:2 -H]-	4.20E+05	2.08	45013376	3.75
480.3096	13.0	LPE	LPE 18:0	[LPE 18:0 -H]-	2.72E+06	13.43	6.79E+08	56.61
452.2783	13.2	LPE	LPE 16:0	[LPE 16:0 -H]-	1.67E+06	8.24	2.82E+08	23.51
776.56	14.9	PCO	PCO 38:6	[PCO 38:6 -CH_3]-	4.94E+06	2.67	3348976	13.72
836.5811	14.9	PCO	PCO 38:6	[PCO 38:6 +HCO_2]-	6.02E+07	32.58	3348976	13.72
834.5654	14.9	PCO	PCO 38:7	[PCO 38:7 +HCO_2]-	1.18E+07	6.39	0	0
820.5862	14.9	PC	PC 40:5	[PC 40:5 -CH_3]-	2.85E+06	0.42	64641805	1.21
880.6073	14.9	PC	PC 40:5	[PC 40:5 +HCO_2]-	4.58E+07	6.74	64641805	1.21
876.576	14.9	PC	PC 40:7	[PC 40:7 +HCO_2]-	8.16E+05	0.12	16882505	0.32
878.5917	14.9	PC	PC 40:6	[PC 40:6 +HCO_2]-	7.05E+06	1.04	1.14E+08	2.14
862.5967	15.0	PCO	PCO 40:7	[PCO 40:7 +HCO_2]-	1.30E+06	0.7	6956098	28.5
852.576	15.0	PC	PC 38:5	[PC 38:5 +HCO_2]-	2.28E+08	33.57	94194919	1.76
792.5549	15.0	PC	PC 38:5	[PC 38:5 -CH_3]-	1.38E+07	2.03	94194919	1.76
790.5392	15.0	PC	PC 38:6	[PC 38:6 -CH_3]-	2.41E+06	0.35	1.35E+08	2.52
850.5604	15.1	PC	PC 38:6	[PC 38:6 +HCO_2]-	3.98E+07	5.86	1.35E+08	2.52
810.5654	15.1	PCO	PCO 36:5	[PCO 36:5 +HCO_2]-	2.31E+06	1.25	474379.8	1.94
908.5811	15.1	PCO	PCO 44:12	[PCO 44:12 +HCO_2]-	9.91E+05	0.54	0	0
824.5447	15.1	PC	PC 36:5	[PC 36:5 +HCO_2]-	1.25E+06	0.18	3758941	0.07
842.5705	15.1	PC	PC 42:8	[PC 42:8 -CH_3]-	4.05E+06	0.6	0	0
826.5604	15.1	PC	PC 36:4	[PC 36:4 +HCO_2]-	8.41E+06	1.24	1.37E+08	2.57
838.5967	15.1	PCO	PCO 38:5	[PCO 38:5 +HCO_2]-	4.21E+07	22.79	3200670	13.11
830.5341	15.2	PCO	PCO 38:9	[PCO 38:9 +HCO_2]-	5.10E+05	0.28	0	0
768.5549	15.2	PC	PC 36:3	[PC 36:3 -CH_3]-	1.09E+06	0.16	1.12E+08	2.1
812.5811	15.2	PCO	PCO 36:4	[PCO 36:4 +HCO_2]-	5.01E+06	2.71	965487.6	3.96
860.5811	15.2	PCO	PCO 40:8	[PCO 40:8 +HCO_2]-	1.29E+06	0.7	0	0
772.5862	15.2	PC	PC 36:1	[PC 36:1 -CH_3]-	1.20E+06	0.18	1.67E+08	3.13
786.5654	15.2	PCO	PCO 34:3	[PCO 34:3 +HCO_2]-	1.05E+06	0.57	421926.4	1.73
770.5705	15.2	PC	PC 36:2	[PC 36:2 -CH_3]-	2.37E+06	0.35	2.81E+08	5.26
828.576	15.3	PC	PC 36:3	[PC 36:3 +HCO_2]-	1.26E+07	1.86	1.12E+08	2.1
832.6073	15.3	PC	PC 36:1	[PC 36:1 +HCO_2]-	1.91E+07	2.81	1.67E+08	3.13
830.5917	15.3	PC	PC 36:2	[PC 36:2 +HCO_2]-	3.29E+07	4.85	2.81E+08	5.26
814.5967	15.3	PCO	PCO 36:3	[PCO 36:3 +HCO_2]-	5.97E+06	3.23	0	0
872.5447	15.3	PC	PC 40:9	[PC 40:9 +HCO_2]-	2.28E+06	0.34	7895566	0.15
816.6124	15.3	PCO	PCO 36:2	[PCO 36:2 +HCO_2]-	4.23E+06	2.29	0	0
744.5549	15.3	PC	PC 34:1	[PC 34:1 -CH_3]-	1.16E+07	1.71	9.73E+08	18.2
760.5498	15.3	PCO	PCO 32:2	[PCO 32:2 +HCO_2]-	4.11E+05	0.22	0	0
742.5392	15.3	PC	PC 34:2	[PC 34:2 -CH_3]-	5.52E+06	0.81	6.61E+08	12.37
804.576	15.3	PC	PC 34:1	[PC 34:1 +HCO_2]-	1.48E+08	21.8	9.73E+08	18.2
802.5604	15.3	PC	PC 34:2	[PC 34:2 +HCO_2]-	6.23E+07	9.18	6.61E+08	12.37
800.5447	15.3	PC	PC 34:3	[PC 34:3 +HCO_2]-	1.79E+06	0.26	21257319	0.4
819.6233	15.4	SM	SM 38:1;3	[SM 38:1;3 +HCO_2]-	9.70E+05	0.18	0	0

787.5759	15.4	SM	SM 42:7;2	[SM 42:7;2 -CH_3]-	1.53E+06	0.28	0	0
788.5811	15.4	PCO	PCO 34:2	[PCO 34:2 +HCO_2]-	5.51E+06	2.98	0	0
728.56	15.4	PCO	PCO 34:2	[PCO 34:2 -CH_3]-	8.43E+05	0.46	0	0
730.5756	15.4	PCO	PCO 34:1	[PCO 34:1 -CH_3]-	1.12E+06	0.6	0	0
776.5447	15.4	PC	PC 32:1	[PC 32:1 +HCO_2]-	7.79E+06	1.15	28607426	0.54
790.5967	15.4	PCO	PCO 34:1	[PCO 34:1 +HCO_2]-	6.04E+06	3.27	0	0
730.5756	15.4	PCO	PCO 34:1	[PCO 34:1 -CH_3]-	1.17E+06	0.64	0	0
718.5392	15.5	PC	PC 32:0	[PC 32:0 -CH_3]-	1.91E+06	0.28	19795245	0.37
778.5604	15.5	PC	PC 32:0	[PC 32:0 +HCO_2]-	1.44E+07	2.11	19795245	0.37
790.5967	15.5	PCO	PCO 34:1	[PCO 34:1 +HCO_2]-	5.90E+06	3.19	0	0
702.5443	15.5	PCO	PCO 32:1	[PCO 32:1 -CH_3]-	6.02E+05	0.33	356250.2	1.46
762.5654	15.5	PCO	PCO 32:1	[PCO 32:1 +HCO_2]-	4.55E+06	2.46	356250.2	1.46
704.56	15.5	PCO	PCO 32:0	[PCO 32:0 -CH_3]-	1.66E+06	0.9	2490120	10.2
764.5811	15.5	PCO	PCO 32:0	[PCO 32:0 +HCO_2]-	1.38E+07	7.48	2490120	10.2
736.5498	15.6	PCO	PCO 30:0	[PCO 30:0 +HCO_2]-	1.26E+06	0.68	0	0
676.5287	15.6	PCO	PCO 30:0	[PCO 30:0 -CH_3]-	1.98E+05	0.11	0	0
929.6753	15.9	SM	SM 48:8;2	[SM 48:8;2 +HCO_2]-	8.82E+05	0.16	0	0
987.7536	16.0	SM	SM 52:7;2	[SM 52:7;2 +HCO_2]-	1.42E+06	0.26	0	0
989.7692	16.0	SM	SM 52:6;2	[SM 52:6;2 +HCO_2]-	8.90E+06	1.61	0	0
901.7168	16.0	SM	SM 50:6;2	[SM 50:6;2 -CH_3]-	7.04E+05	0.13	0	0
961.7379	16.0	SM	SM 50:6;2	[SM 50:6;2 +HCO_2]-	1.22E+07	2.2	0	0
959.7223	16.0	SM	SM 50:7;2	[SM 50:7;2 +HCO_2]-	1.61E+06	0.29	0	0
963.7536	16.0	SM	SM 50:5;2	[SM 50:5;2 +HCO_2]-	8.01E+06	1.45	0	0
933.7066	16.0	SM	SM 48:6;2	[SM 48:6;2 +HCO_2]-	4.90E+06	0.89	0	0
935.7223	16.1	SM	SM 48:5;2	[SM 48:5;2 +HCO_2]-	5.26E+07	9.49	0	0
847.6698	16.1	SM	SM 46:5;2	[SM 46:5;2 -CH_3]-	2.81E+06	0.51	0	0
905.6753	16.1	SM	SM 46:6;2	[SM 46:6;2 +HCO_2]-	4.32E+05	0.08	0	0
907.691	16.1	SM	SM 46:5;2	[SM 46:5;2 +HCO_2]-	3.69E+07	6.66	0	0
821.6542	16.1	SM	SM 44:4;2	[SM 44:4;2 -CH_3]-	4.68E+05	0.08	347492.8	0.02
879.6597	16.2	SM	SM 44:5;2	[SM 44:5;2 +HCO_2]-	2.00E+06	0.36	1379689	0.09
881.6753	16.2	SM	SM 44:4;2	[SM 44:4;2 +HCO_2]-	6.01E+06	1.09	347492.8	0.02
885.7066	16.2	SM	SM 44:2;2	[SM 44:2;2 +HCO_2]-	8.67E+05	0.16	201169.4	0.01
887.7223	16.2	SM	SM 44:1;2	[SM 44:1;2 +HCO_2]-	4.99E+05	0.09	0	0
859.691	16.2	SM	SM 42:1;2	[SM 42:1;2 +HCO_2]-	2.04E+07	3.68	11508323	0.72
799.6698	16.2	SM	SM 42:1;2	[SM 42:1;2 -CH_3]-	2.15E+06	0.39	11508323	0.72
857.6753	16.2	SM	SM 42:2;2	[SM 42:2;2 +HCO_2]-	2.95E+07	5.33	37121190	2.34
797.6542	16.2	SM	SM 42:2;2	[SM 42:2;2 -CH_3]-	2.79E+06	0.5	37121190	2.34
855.6597	16.2	SM	SM 42:3;2	[SM 42:3;2 +HCO_2]-	3.76E+06	0.68	19111127	1.2
771.6385	16.2	SM	SM 40:1;2	[SM 40:1;2 -CH_3]-	1.61E+06	0.29	17473410	1.1
845.6542	16.3	SM	SM 46:6;2	[SM 46:6;2 -CH_3]-	1.75E+06	0.32	0	0
831.6597	16.3	SM	SM 40:1;2	[SM 40:1;2 +HCO_2]-	1.58E+07	2.86	17473410	1.1
877.7015	16.3	SM	SM 42:0;3	[SM 42:0;3 +HCO_2]-	3.28E+06	0.59	0	0

829.644	16.3	SM	SM 40:2;2	[SM 40:2;2 +HCO_2]-	2.99E+06	0.54	0	0
743.6072	16.3	SM	SM 38:1;2	[SM 38:1;2 -CH_3]-	4.96E+06	0.9	0	0
873.6702	16.3	SM	SM 42:2;3	[SM 42:2;3 +HCO_2]-	2.12E+06	0.38	1347243	0.08
803.6284	16.3	SM	SM 38:1;2	[SM 38:1;2 +HCO_2]-	4.04E+07	7.29	0	0
849.6702	16.3	SM	SM 40:0;3	[SM 40:0;3 +HCO_2]-	1.63E+06	0.29	0	0
817.6076	16.3	SM	SM 38:2;3	[SM 38:2;3 +HCO_2]-	2.28E+06	0.41	0	0
813.6491	16.4	SM	SM 42:2;3	[SM 42:2;3 -CH_3]-	1.66E+05	0.03	1347243	0.08
715.5759	16.4	SM	SM 36:1;2	[SM 36:1;2 -CH_3]-	1.57E+07	2.83	47451881	2.99
773.5814	16.4	SM	SM 36:2;2	[SM 36:2;2 +HCO_2]-	9.51E+05	0.17	5689401	0.36
775.5971	16.4	SM	SM 36:1;2	[SM 36:1;2 +HCO_2]-	8.99E+07	16.24	47451881	2.99
713.5603	16.4	SM	SM 36:2;2	[SM 36:2;2 -CH_3]-	1.66E+05	0.03	5689401	0.36
618.3776	16.5	LPC	LPC 22:3	[LPC 22:3 +HCO_2]-	5.47E+04	1.01	0	0
817.5501	16.5	SM	SM 40:8;2	[SM 40:8;2 +HCO_2]-	4.08E+05	0.07	1133054	0.07
747.5658	16.5	SM	SM 34:1;2	[SM 34:1;2 +HCO_2]-	1.25E+08	22.49	6.55E+08	41.23
687.5446	16.5	SM	SM 34:1;2	[SM 34:1;2 -CH_3]-	2.43E+07	4.39	6.55E+08	41.23
703.5396	16.5	SM	SM 34:1;3	[SM 34:1;3 -CH_3]-	9.42E+05	0.17	1254347	0.08
745.5501	16.5	SM	SM 34:2;2	[SM 34:2;2 +HCO_2]-	1.23E+06	0.22	704893.6	0.04
763.5607	16.5	SM	SM 34:1;3	[SM 34:1;3 +HCO_2]-	5.11E+06	0.92	1254347	0.08
721.5501	16.5	SM	SM 32:0;2	[SM 32:0;2 +HCO_2]-	1.54E+06	0.28	0	0
685.529	16.5	SM	SM 34:2;2	[SM 34:2;2 -CH_3]-	4.09E+05	0.07	704893.6	0.04
705.5552	16.6	SM	SM 34:0;3	[SM 34:0;3 -CH_3]-	3.16E+05	0.06	0	0
659.5133	16.6	SM	SM 32:1;2	[SM 32:1;2 -CH_3]-	1.88E+06	0.34	5351894	0.34
719.5345	16.6	SM	SM 32:1;2	[SM 32:1;2 +HCO_2]-	7.05E+06	1.27	5351894	0.34
614.3463	16.8	LPC	LPC 22:5	[LPC 22:5 +HCO_2]-	1.24E+06	22.93	1668564	0.1
612.3307	16.9	LPC	LPC 22:6	[LPC 22:6 +HCO_2]-	1.97E+05	3.65	4425346	0.27
550.3514	16.9	LPCO	LPCO 18:2	[LPCO 18:2 +HCO_2]-	5.75E+04	2.26	0	0
588.3307	16.9	LPC	LPC 20:4	[LPC 20:4 +HCO_2]-	7.34E+04	1.36	7767312	0.48
566.3463	17.1	LPC	LPC 18:1	[LPC 18:1 +HCO_2]-	3.11E+05	5.76	1.24E+08	7.59
506.3252	17.1	LPC	LPC 18:1	[LPC 18:1 -CH_3]-	2.25E+05	4.17	1.24E+08	7.59
568.362	17.2	LPC	LPC 18:0	[LPC 18:0 +HCO_2]-	5.97E+05	11.05	2.53E+08	15.53
508.3409	17.2	LPC	LPC 18:0	[LPC 18:0 -CH_3]-	3.84E+05	7.11	2.53E+08	15.53
492.3459	17.2	LPCO	LPCO 18:1	[LPCO 18:1 -CH_3]-	1.36E+05	5.34	748277.1	28.56
480.3096	17.4	LPC	LPC 16:0	[LPC 16:0 -CH_3]-	1.18E+06	21.78	4.31E+08	26.45
540.3307	17.4	LPC	LPC 16:0	[LPC 16:0 +HCO_2]-	1.14E+06	21.17	4.31E+08	26.45
492.3459	17.6	LPCO	LPCO 18:1	[LPCO 18:1 -CH_3]-	3.25E+05	12.76	748277.1	28.56
552.3671	17.6	LPCO	LPCO 18:1	[LPCO 18:1 +HCO_2]-	3.45E+05	13.56	748277.1	28.56
466.3303	17.7	LPCO	LPCO 16:0	[LPCO 16:0 -CH_3]-	4.01E+05	15.75	0	0
526.3514	17.8	LPCO	LPCO 16:0	[LPCO 16:0 +HCO_2]-	2.99E+05	11.73	0	0

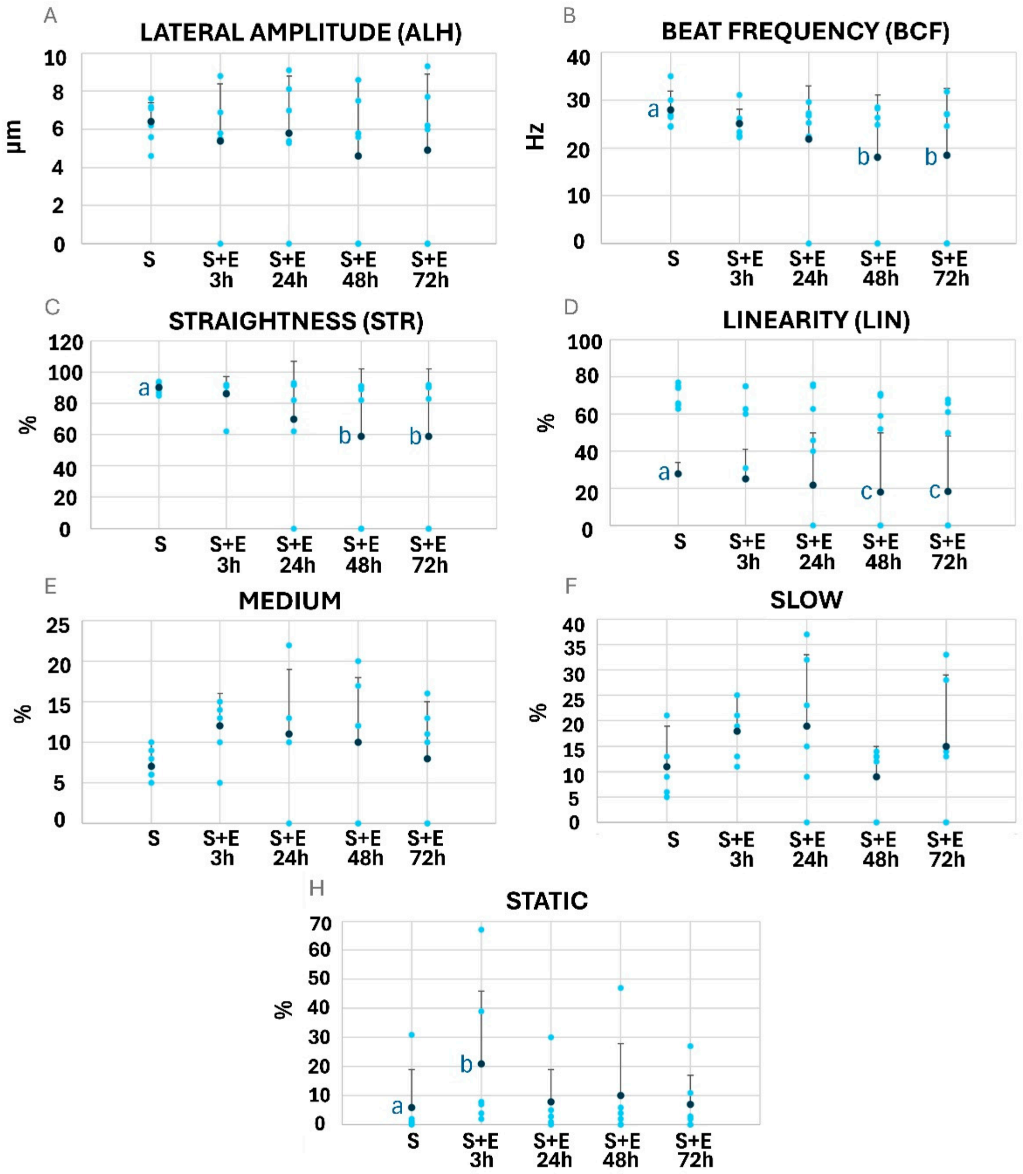

Figure S1. Computer-assisted sperm analysis was performed on fresh semen and on semen stored at 4 °C in TRIS–egg yolk extender for up to 72 h. The figure reports 7 representative functional parameters: lateral amplitude (ALH; A), beat frequency (BCF; B), straightness (STR; C), linearity (LIN; D), percentage of medium spermatozoa (E), percentage of slow spermatozoa (F), and percentage of static spermatozoa (H). Data are shown as mean ± SEM; individual points represent single dogs ($n = 6$). Statistical differences among time points were assessed by repeated-measures one-way ANOVA followed by Tukey's multiple-comparison test, considering the dog as the repeated subject. Significance levels are indicated as $p < 0.05$ (a,b), and p < 0.01 (a,c). Fresh, freshly collected semen; S+E, semen diluted with TRIS–egg yolk extender and stored at 4 °C for the indicated time. S, untreated semen at the initial time point; S+E, semen diluted with TRIS–egg yolk extender and stored at 4 °C for the indicated time.
